# Osteoradionecrosis of the Hip, a Troublesome Complication of Radiation Therapy: Case Series and Systematic Review

**DOI:** 10.3389/fmed.2022.858929

**Published:** 2022-03-25

**Authors:** Sheng-hao Xu, Jin-shuo Tang, Xian-yue Shen, Zhi-xin Niu, Jian-lin Xiao

**Affiliations:** ^1^Department of Orthopedics, China-Japan Union Hospital of Jilin University, Changchun, China; ^2^Department of Orthopedics, The Second Hospital of Jilin University, Changchun, China

**Keywords:** osteoradionecrosis, hip, cancer, lymphoma, case report

## Abstract

**Background:**

Osteoradionecrosis of the hip is a serious complication of radiotherapy that is easily overlooked by physicians and patients in the early stages. There are relatively few reports on this subject, so there is no clear scientific consensus for the pathogenesis, early diagnosis, and clinical treatment of hip osteoradionecrosis. In this paper, we report two cases of hip osteoradionecrosis and systematically review the related literature.

**Case Presentation:**

We report two cases of hip osteoradionecrosis. One patient successfully underwent total hip arthroplasty in our hospital and recovered well postoperatively. Another patient although we offered a variety of surgical options for this patient, the patient was worried that the bone loss would lead to poor prosthesis fixation, resulting in prosthesis loosening and infection, and therefore ultimately refused surgical treatment.

**Conclusion:**

With the development of radiological techniques, the incidence of hip osteoradionecrosis is decreasing year by year, but early diagnosis and rational treatment remain challenging. The effects of non-surgical treatment are limited. Early prevention, early detection, and early intervention are crucial to delay or prevent the emergence of more serious complications.

## Introduction

Osteoradionecrosis (ORN) is an area of bare inactivated irradiated bone that fails to heal within 3–6 months despite the absence of local tumor disease (1–5). ORN is also called radiotherapy-induced osteonecrosis, aseptic necrosis, or avascular necrosis (6), and is caused by lack of blood supply to the bones, which ultimately leads to ischemic cell death (7). In response to ORN, there is an increase in the osteoclast activity in an attempt to remove the necrotic bone as well as an increase in the osteoblast activity to repair the resulting damage; eventually, however, the bone structure collapses. Certain bones like the mandible and pelvis seem to be particularly susceptible to ORN (8). The sacrum is the most common bone affected by pelvic radiation because it has a large amount of red bone marrow and is located at the center of the irradiated area (9). The incidence of pelvic ORN varies widely, ranging from 2.1 to 34%, depending on the radiotherapy technology and standards applied (10–12). In the case of the proximal femur, ORN leads to progressive pain that is exacerbated by weight-bearing and results in the loss of joint function. Moreover, ORN of the hip has been well described as a complication of radiotherapy (13, 14).

The side effects of radiotherapy on the bone are critical but underappreciated issue in clinical practice (15, 16). ORN of the hip is a rare disease, which needs to be highly suspected. There are few cases of ORN of the hip recorded in the literature. The objective of the present systematic review is to diagnose and manage this rare disease by analyzing two cases in our department and systematically reviewing relevant literature, and to explore the clinical manifestations, diagnosis and treatment and related preventive measures of ORN, so as to prevent delayed diagnosis and reduce pain and disability of the patients.

## Case Presentation

### Case 1

**FIGURE 1:**
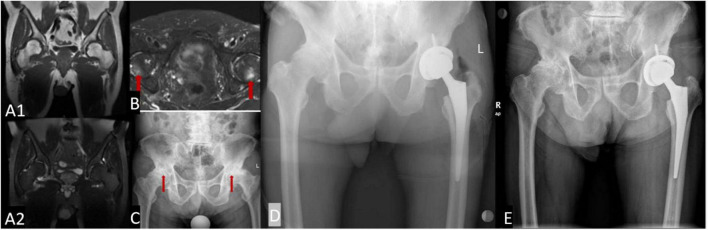
The morphology of the acetabulum and femoral head is bilaterally normal, with uniform internal signals **(A1,A2)**; when underwent 3 months of radiotherapy, femoral head with slightly longer T1 and T2 signals, and uneven and patchy internal signals **(B)**. Bilateral hip joint space narrowing is seen on X-ray examination, and the articular surface is less smooth. The femoral heads on both sides have become flattened and show uneven density **(C)**. Post-operative examination an artificial hip replacement is seen on the left hip, which is in a normal position **(D)**. Two and a half years after surgery, the prosthesis is in normal position **(E)**.

An 83-year-old man presented with bilateral hip pain and limited mobility for more than 2 years. The restriction of movement had been progressive and had become worse during the past 6 months. During the past 2 years, he had lost 10 kg without deliberately trying to lose weight. He had no history of trauma, and reported no obvious inducement for the limitation of joint movement. In 2012, he had undergone magnetic resonance imaging (MRI) examination of both hips, which showed no obvious abnormalities (Figures 1A1,A2). In 2015, he was diagnosed with prostate cancer, and underwent 3 months of radiotherapy. He developed radiation enteritis and blood in the stools during radiotherapy, and was symptomatically treated with a compound carrageenan suppository. Shortly after the radiotherapy, he began to experience difficulty in walking and intermittent lameness. An MRI of both hips taken 3 months after radiotherapy showed bilateral ORN of the femoral head (Figure 1B).

The patient was admitted to our hospital, where a physical examination revealed no obvious redness or swelling around the hip joints. The skin temperature was normal, and there was slight tenderness around the hip joints. Hip movements were bilaterally limited. The FABER test was bilaterally positive (+). Bilateral hip joint X-ray examination was performed during the present hospitalization, and it showed bilateral joint space narrowing and flattening of the femoral head (Figure 1C). Considering the patient’s medical history, laboratory test results, imaging findings, and physical examination findings, we diagnosed the patient with bilateral ORN of the hip joint. The patient underwent left total hip arthroplasty (THA) (Figure 1D). Due to the advanced age of the patient, the symptoms of the right hip joint were less severe than those of the contralateral hip joint, so the right hip was treated conservatively.

After 2.5 years of follow-up, the patient’s general condition remained good, a recurrence of radiation enteritis during the follow-up period. The activity of the left lower limb was significantly improved, and the joint pain had disappeared; however, the movement of the right lower limb was still limited (Figure 1E).

### Case 2

A 65-year-old man presented with a 10-year history of pain in the left hip and a 3-month history of worsening of pain after activity. The patient did not report any obvious cause for left hip pain, limited mobility, and abnormal gait. Ten years ago, he had been diagnosed with lymphoma at a local hospital, and had received regular chemotherapy and radiotherapy.

**FIGURE 2:**
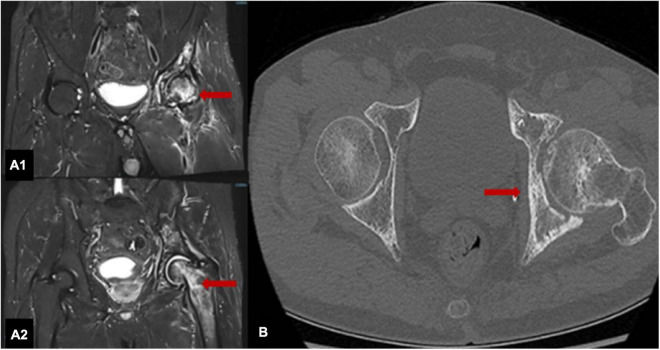
MRI scans of both hips show that the shapes of the left femoral head and upper femur are normal, with patchy T1 and T2 signals, uneven signals and patchy low-signal areas; the right hip joint adjacent bone shows uniform signals **(A1,A2)**. The left hip joint is narrow, and the articular surface is not smooth; the left femoral head is slightly flattened with a patchy high-density shadow with an unclear boundary **(B)**.

A specialist physical examination during the present hospitalization revealed no obvious redness over the left hip, normal skin temperature, tenderness in the left hip joint (+), limited movement of the left hip joint, positive FABER test (+) for the left hip joint, and grade IV muscle strength in the left lower limb. There was no enlargement of the superficial lymph nodes. Bilateral hip joint MRI (Figures 2A1,A2) examinations and computed tomography (CT) (Figure 2B) showed necrosis of the left femoral head accompanied with the bone loss and destruction. ORN of the left hip was diagnosed based on medical history and imaging findings.

Although we recommended surgical options that could overcome possible poor fixation (for example long stems, triflange cups, megaprosthesis, etc.) and allow the patient to be treated, the family of the patient ultimately declined surgical treatment due to concerns about the bone loss and the risk of possible loosening of the prosthesis and infection shortly after surgery. We therefore recommended that patient take conservative treatment measures such as fixed point, quantitative radiotherapy, and anti-osteoporosis drugs.

The results of the follow-up were not very satisfactory, and the family members of the patient did not want us to understand the true condition of the patient so that we could not evaluate the final result of the patient.

## Materials and Methods

### Search Strategy and Eligibility Criteria

A systematic review was performed according to the PRISMA (Preferred Reporting Items for Systematic Reviews and Meta-Analyses) guidelines. We conduct literature searches of the PubMed, Embase, and Web of Science databases for case reports of ORN of the hip joint published between month date, 1980 and December 31, 2020. The following search terms are used for the literature searches: PubMed, [(osteoradionecrosis) OR (radiation necrosis)] AND [(hip) OR (femoral head)]; Embase, {“osteoradionecrosis”/exp OR osteoradionecrosis OR [(“radiation”/exp OR radiation) AND (“necrosis”/exp OR necrosis)]} AND {“hip”/exp OR hip OR [femoral AND (“head”/exp OR head)]}; and Web of Science, TS = (osteoradionecrosis OR radiation necrosis) AND TS = (hip OR femoral head).

The inclusion criteria were: (1) access to patient-related information; (2) case report series; (3) hips exclude the tumor metastasis. (4) hips with a history of radiation. The exclusion criteria were: (1) non-English language; (2) unable to obtain patient-related information; (3) off-topic; (4) unidentifiable due to the tumor metastasis.

### Data Extraction

Data extraction from each study was performed independently by first author and then reviewed by author JLX. Included literatures were further analyzed and classified. The useful data variables of the 25 published cases and the two present cases of ORN of the hip are listed in Table 1.

**TABLE 1:** Clinical characteristics of osteoradionecrosis of the hip.

References	Year	Age	Sex	Chief complaints	Primary disease necessitating radiation	Total radiation dose	Necrosis site	Management	Outcome
Thorne et al. (21)	1981	37	F	Stiffness in both thighs and pain in the groin	Hodgkin disease	35 Gy	Right hip	Conservative	Symptoms resolved
Nobler (22)	1984	53	F	Pain and limitation of motion	Epidermoid carcinoma of the cervix	Not clear	Both hips	Conservative	Death
Csuka et al. (23)	1987	73	M	Pain	Prostatic cancer	Not clear	Both hips	Not clear	Not clear
Deleeuw and Pottenger (24)	1988	63	F	Pain	Squamous cell carcinoma of the cervix	67 Gy	Both hips	Left: total hip arthroplasty Right: conservative	Mild to moderate pain
		50	M	Pain	Squamous cell carcinoma of the anus	50 Gy	Both hips	Bilateral total hip arthroplasty	Walks well
Phillips and Rao (25)	1989	68	F	Pain	Adenocarcinoma of the uterus	50 Gy	Both hips	Bilateral hip replacement	Condition deteriorated
		74	F	Pain	Adenocarcinoma of the uterus	50 Gy	Right hip	Right hip replacement	Walks well
		48	F	Pain	Adenocarcinoma of the uterus	50 Gy	Right hip	Right hip replacement	Walks well
		82	F	Pain	Adenocarcinoma of the cervix	51.4 Gy	Left hip	Left hip replacement	Walks well
Jenkins et al. (18)	1995	66	F	Discomfort in left groin	Squamous cell carcinoma of the anus	49.5 Gy	Both hips	Left: excision of the femoral head Right: conservative	Not clear
		65	M	Pain	Squamous cell carcinoma of the anus	48 Gy	Left hip	Dynamic hip screw	Regained reasonable mobility
		61	F	Diminished mobility and pain	Squamous cell carcinoma of the vulva	45 Gy	Both hips	Bilateral total hip arthroplasty	Not clear
		59	F	Pain and limitation of motion	Squamous carcinoma of the vulva	45 Gy	Both hips	Bilateral hemiarthroplasty	Not clear
Boudreau et al. (26)	1999	Not clear	Not clear	Pain	Vulvar squamous cell carcinoma	Not clear	Right hip	Hip replacement	Uneventful
Dhadda and Chan (20)	2006	41	F	Pain	Squamous cell carcinoma of the cervix	45 Gy	Both hips	Bilateral total hip arthroplasty	Not clear
Goitz et al. (27)	2007	61	M	Pain and limitation of motion	Proximal femur adenoma	Not clear	Left hip	Total hip arthroplasty	Fully ambulatory
Quinlan et al. (28)	2009	67	M	Pain	Squamous cell carcinoma of the urethra	50 Gy	Both hips	Bilateral hip arthroplasty	Walks well
Chung et al. (29)	2010	78	F	None	Squamous cell carcinoma of the vagina	Not clear	Right hip	None	Symptom-free
Michalecki et al. (30)	2011	65	F	Pain	Cervical cancer	44 Gy	Right hip	Curettage of the joint with bone graft	Not clear
		70	M	Femoral neck fracture	Urinary bladder, urothelial carcinoma	66 Gy	Left hip	Total hip arthroplasty	Not clear
Vuong et al. (31)	2012	60	M	Pain	Adenocarcinoma of the sigmoid colon	Not clear	Left hip	Total hip arthroplasty	Not clear
Abdulkareem (32)	2013	74	M	Pain	Prostate cancer	Not clear	Left hip	Total hip arthroplasty	Uneventful
Win and Aparici (33)	2015	63	M	Pain	Prostate cancer	Not clear	Right hip	Not clear	Not clear
Daoud et al. (34)	2016	51	M	Pain	Prostate cancer	Left: 8.8 Gy; Right: 15.3 Gy	Left hip	Conservative	Death
Nagi et al. (35)	2018	72	F	Pain	Adenocarcinoma of the endometrium	Not clear	Right hip	Total hip arthroplasty	Fully mobile hip
Our cases	2021	83	M	Pain and limitation of motion	Prostate cancer	Not clear	Both hips	Left: total hip arthroplasty Right: Conservative	Left: Uneventful Right: Motion limitation
		65	M	Pain	Lymphoma	Not clear	Left hip	Conservative	Lost to follow-up

### Methodological Quality Assessment

The methodological quality of the studies included was assessed using the quality assessment tool of case report (17). All articles were assessed independently by two authors (XYS and JST), and any disagreements regarding the quality assessment were resolved by discussion and consensus involving a senior reviewer (JLX).

## Results

**FIGURE 3:**
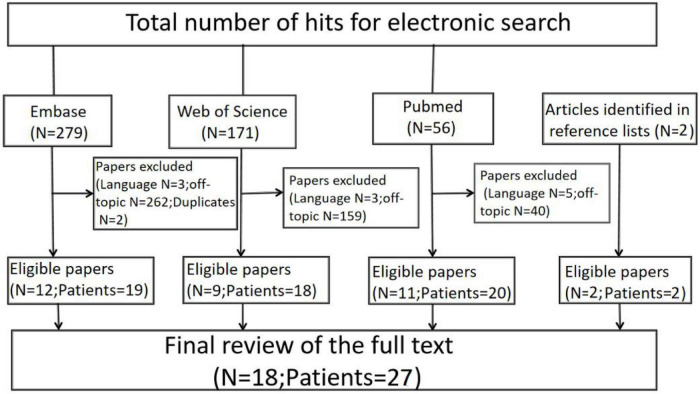
Flow chart depicting the systematic review process used in this study.

The reference lists of the retrieved papers are also searched to identify further research. The literature on hip ORN thus far mainly consists of case series and case reports (18–35). We review case reports published between 1980 and 2020. A flow diagram of the selection process is shown in Figure 3. The literature searches identify a total of 18 papers involving 28 cases; of these, 3 cases are not relevant. In addition to our current 2 cases, we finally obtained a total of 27 related case information.

### Characteristics of the Included Studies

According to the statistics of the included cases, except for 1 case of unreported age, the minimum reported age was 37 years old, the maximum age was 83 years old, and the average age was 63 years old. One case did not record patient symptoms, 1 patient presented with joint discomfort, 1 patient presented with femoral neck fracture, and joint pain was present in all remaining patients, 6 patients of whom also had limited joint motion. Among the 27 cases, 3 of these did not report the treatment modality, and the remaining 24 cases included 33 diseased hips, 7 of the 33 hips which were treated conservatively, with only one case symptomatic relief and the rest having poor outcomes; 23 of the 26 hips were treated with arthroplasty. Among these, 12 hips of which recovered well, 8 hips had unclear outcomes, and the remaining 3 hips had persistent or worsening symptoms. The clinical features of the 27 cases of ORN of the hip are listed in Table 1.

**FIGURE 4:**
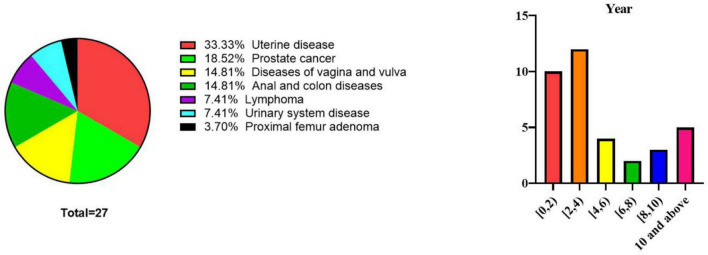
Distribution of diseases that necessitated radiotherapy in all 27 patients and time interval between radiotherapy and onset of osteoradionecrosis (In cases of bilateral hip joint osteoradionecrosis, each joint was counted separately: *n* = 36 cases, excluding 2 cases with no reported interval time).

The most common primary diseases that necessitated radiotherapy were uterine, prostate, vulvar, and anal cancers and more than half of the patients were diagnosed with ORN of the hip on the basis of symptoms or radiographic findings within 4 years of undergoing radiotherapy (Figure 4).

### Quality Assessment of Case Report

According to the evaluation items on the quality assessment tool of case report (17), 92.6% of the cases included in this article clearly described the demographic characteristics of the patients; 92.6% of the cases clearly described the patient’s medical history in chronological order; 88.9% of the cases clearly described the patient’s current clinical presentation; 62.9% of the cases clearly describe the method of diagnosis or evaluation and the results; 88.9% of the cases clearly described the intervention or treatment measures; 62.9% of the cases clearly described the clinical manifestations after treatment; 22.2% of the cases identified and described adverse reactions or accidents; 100% of the cases provided reference experience. To sum up, all included studies were considered to be of moderate or high quality.

## Discussion

Radiotherapy is one of the most effective treatments for cancer, and can be used to effectively control local disease and provide palliative care (36). Radiotherapy targets all cells with high turnover rates, regardless of whether they are malignant tumor cells or normal host tissue cells. Therefore, a balance between tumor eradication and normal tissue protection is essential to achieve healing after radiotherapy without weakening the patient (37). Theoretically, bones are relatively slowly proliferating tissues, and show relatively high resistance to ionizing radiation. However, it has been reported that high radiation doses (such as those used for cancer treatment) can greatly increase the risk of ORN (38, 39). This is due to the high calcium content of bone tissue, which absorbs 30–40% more radiation than the surrounding tissue, making bones a common site of radiation injury (40). It has been suggested that the sensitivity of bone cells to radiation damage may be attributable to the effects of chemotherapy drugs; however, the long-term side effects of combination chemotherapy are poorly understood (18, 20).

### Mechanism

#### Vascular Injury

At present, there is no consensus regarding the mechanism of ORN. Early theories suggested that ORN was a manifestation of radiation osteomyelitis (infection), which formed the basis for its treatment with antibiotics (41). In 1926, Ewing described radiation-related skeletal changes as “radiation osteitis” (42), and reported that these consisted of progressive occlusive endarteritis and periarteritis characterized by vascular endothelial cell swelling, cytoplasmic vacuolization, and vascular wall necrosis, which resulted in vascular lumen stenosis, bone marrow hypoxia, and sclerosing transformation (43). After irradiation, the normal and orderly interaction between bone formation and bone resorption is lost, resulting in delayed and weakened remineralization. Consistent with this, in 1983, Marx proposed that radiotherapy would cause a series of events at the cellular and extracellular levels, leading to reduced tissue vascularization, increased hypoxia, and cell damage, which would eventually lead to a chronically non-healing wound (1). Marx also reported that no infection was present in such wounds. According to this theory, persistent hypoxia is the main component of ORN, and this informed the use of hyperbaric oxygen therapy to treat ORN (1).

Radiation-induced vascular damage is a complex process, involving arterial and capillary damage, but veins are less sensitive to radiotherapy (44). In general, this process begins with progressive endothelial cell loss. In animal models, approximately 15% of the endothelium is lost within 24 h after 5–200 Gy irradiation, followed by further endothelial loss that lasts for several months (45). Apoptosis promotes this process in a dose-dependent manner (46), and is mediated by acid sphingomyelinase (47). After the initial loss of endothelial cells, thrombosis and hemorrhage occur in a partially overlapping manner, and are followed by long-term morphological changes, including endothelial proliferation, basement membrane thickening, outer membrane fibrosis, and vasodilation (48), which are important components of vascular remodeling. Large blood vessels develop atherosclerosis, thromboembolism, rupture, and aneurysm. Radiation-induced atherosclerosis often involves plaques that are rich in macrophages, lipids, and areas of bleeding, which differentiate it from classic atherosclerosis (49). Inflammation, increased permeability, and thrombosis occur in small blood vessels, leading to the loss of capillaries and avascular necrosis of the peripheral nerve tissue. The femoral head, humeral head, and proximal parts of the scaphoid and talus are particularly prone to avascular necrosis, because these bones are located at the farthest ends of the skeletal vascular system, with limited access to local blood vessels (50, 51).

Contradicting the above reports, Sengupta and Prathap (52) found few cells and no changes in blood vessels in human bone sections after irradiation. They also demonstrated that osteoblasts were generally more sensitive to radiation than osteoclasts, and radiotherapy was followed by a decrease in the number of osteoblasts, a decrease in collagen production, and a decrease in alkaline phosphatase activity, affecting matrix mineralization (52).

#### Radiation-Induced Fibrosis

Delanian and Lefaix (53) proposed that ORN was attributable to radiation-induced fiber atrophy, which involved free radical formation, endothelial dysfunction, inflammation, microvascular thrombosis, fibrosis, remodeling, and ultimately, bone and tissue necrosis. Ergün and Howland (43) also believed that the main effect of radiation on bone was atrophy, which would lead to a decrease in the number of functional structural components in the tissue (43).

Recent advances in cell and molecular biology have enabled accurate assessments of the progression of ORN under a microscope, and the findings support the theory of radiation-induced fibrosis. According to this mechanism, a series of pathological changes occur, and these can be divided into three stages (53): (i) fibrosis stage: mainly observed in endothelial cells, accompanied by acute inflammation, (ii) histological stage: abnormal fibroblast activity and loss of extracellular matrix organization, and (iii) late fiber atrophy stage: an attempt to reshape the tissue by forming fragile healing tissue. When the tissue suffers local damage, the risk of reactivating tissue inflammation increases, which may lead to tissue necrosis.

### Epidemiology

Since no comprehensive epidemiological studies have been conducted in recent years, the estimated incidence rate of hip ORN is unclear. Dzik-Jurasz et al. (54) reported that femoral head necrosis occurred in 4 of their 763 treated cases. However, Massin and Duparc reviewed 71 hip joints treated with radiotherapy after gynecological cancer, and found that theater of osteonecrosis was 24% (19). The threshold radiation dose that causes bone changes was considered to be 30 Gy, and cell death usually occurs at a dose of 50 Gy (8). Emami et al. proposed radiotherapy guidelines on potentially toxic doses, and recommended a tolerable dose of 52 Gy for normal tissues (55); this recommendation was consistent with the findings of previous studies, which found that only minor changes occur at doses in the range of 42–45 Gy (56). It must be emphasized that this standard radiation dose threshold may not be applicable to patients undergoing concomitant chemotherapy (20).

A historical series reported that the incidence of ORN varied from 2 to 22% (57–59). According to Clayman, the application of ultra-high-pressure radiotherapy significantly reduced the overall prevalence of ORN from 11.8% before 1968 to 5.4% after 1968 (60). Wahl described similar results, noting that the prevalence of ORN was 3% between 1997 and 2006 (61).

### Diagnosis

Histology is the gold standard for the diagnosis of ORN. On histological examination, the condition manifests itself as obvious bone cell destruction, absence of osteoblasts at the bone margins, absence of new osteoid, and atrophic bone tissue changes similar to atrophic changes in the skin or mucosa (6). However, histological examination is usually unnecessary, and is not routinely performed. Furthermore, because of the rarity of this disease, clinicians tend to ignore this serious complication, so although early diagnosis is particularly important, it remains challenging at present. The diagnosis of ORN requires multiple considerations, including the history of radiotherapy, clinical manifestations, and findings of physical examination and imaging studies, such as X-ray examination and MRI (62). The imaging findings depend on the stage and extent of the lesion (63). In the early stages, the density of the affected area increases, which is made more prominent by the osteoporosis of the surrounding tissue; cystic changes appear due to absorption of the dead bone tissue, and varying degrees of collapse can be seen (63). If treatment is provided at this stage, recovery is possible before femoral head collapse occurs; however, without treatment, this process almost inevitably leads to gradual deterioration, which eventually leads to irreversible joint destruction and loss of hip function (64). Over the decades, many theories about the origin of ORN have been proposed. Despite the controversy, most authors agree that the prerequisites for diagnosing ORN are as follows: (i) a history of radiation to the area of bone damage, (ii) no recurrent tumor, (iii) mucosal rupture or failure of healing, resulting in bone exposure, and (iv) necrosis of the covered bone. Pathological fracture, fistula formation, and cellulitis are not necessary for diagnosis (65).

### Differential Diagnosis

It is necessary to distinguish ORN from other hip joint diseases with similar clinical symptoms, such as hip arthritis, bone tumors, and ankylosing spondylitis affecting the hip joint (Table 2) (66). Due to the similar characteristics of ORN and bone metastasis, X-ray examinations are usually not enough to make a diagnosis (67–69). CT and MRI are effective diagnostic tools (68). If these do not yield conclusive results, positron emission tomography can be used (70). Bone imaging shows a typical symmetrical uptake pattern in ORN, while asymmetry is observed in the case of bone metastasis (71, 72).

**TABLE 2:** Differential diagnosis of ORN of the hip (66).

Disease	Age predilection	Sex predilection	Etiology	Unilateral or bilateral	Acetabular involvement	Diagnosis elements
ORN of the hip	Adults and the elderly	No gender differences	Radiation	Bilateral	Yes	History of radiation therapy; MRI: acetabulum, pubis, femoral head, and upper femur show long T1 and T2 signals.
Osteoarthritis	Middle-aged and older	No gender differences	Degeneration	Bilateral	Yes	CT: sclerotic bone and cystic change; MRI: crescent sign
Secondary acetabular dysplasia	Children and youth	Female	Genetic factors	Bilateral	Yes	X-rays: hip joint dislocation, hip joint space narrowing, and features of secondary osteoarthritis
Ankylosing spondylitis involving the hip	Teenagers	Male	Genetic and environmental factors	Bilateral	Yes	HLA-B27(+), sacroiliac joint erosions, and iliac subchondral sclerosis
Idiopathic transient osteoporosis of the hip	Middle-aged and youth	No gender differences	None	Unilateral	No	MRI: low signal intensity on T1WI, high signal intensity on T2WI, extending from the femoral head to the intertrochanteric region
Chondroblastoma of the femoral head	Children and teenagers	Male	Unclear	Unilateral	No	MRI: high signal intensity on T2WI; CT: irregular bone dissolution
Subchondral insufficiency fracture	Elderly	Female	Osteoporosis	Unilateral	No	X-rays: flattening of the femoral head; MRI: subchondral low signal intensity on T1WI and T2WI, with bone marrow edema
Pigmented villonodular synovitis	Young adults	No gender differences	None	Unilateral	Yes	X-rays and CT: hip joint space narrowing; MRI: extensive thickening of the joint lining or an extensive mass, possibly with destructive bone changes
Bone infarction	Unclear	Unclear	Unclear	Bilateral	No	MRI: high signal intensity on T2WI, characteristic double-line sign, which consists of a hyperintense inner ring and a hypointense outer ring

### Therapy

The treatment of ORN of the hip can be categorized into non-surgical and surgical treatments. Non-surgical treatments include restriction of weight-bearing during exercise, hyperbaric oxygen therapy, drug therapy, and biophysical therapy, but the effects are limited (73, 74). Surgical treatment mainly consists of THA.

#### Non-surgical Management

Hyperbaric oxygen therapy seems to be a useful adjuvant treatment for radiation necrosis of the bone and soft tissues. By increasing the supply of hyperbaric oxygen to the relatively non-vascularized and hypoxic radiation-induced necrotic wounds, it is possible to stimulate collagen production and angiogenesis, thereby promoting healing (75). Animal studies have shown that oxygen levels in the hypoxic areas of chronic wounds need to be close to 24–30 mm Hg to produce optimal collagen levels and new blood vessels, and hyperbaric oxygen is usually necessary to achieve this result (76, 77). Moreover, hyperbaric oxygen therapy has bactericidal or antibacterial effects (78).

However, since the Delanian theory of fiber atrophy was proposed, it has been widely accepted as the main mechanism of ORN, which opens the door to the use of antioxidants and anti-fibrotic drugs as the main treatment options for ORN (60, 79, 80). These drugs include pentoxifylline, tocopherol, and clodronate (80). Pentoxifylline can induce blood vessel dilation, increase red blood cell elasticity, and increase blood flow. It also has anti-tumor necrosis factor α activity, and is believed to reduce the cytokine cascade that drives the progression of ORN (81). Although pentoxifylline can improve the symptoms of peripheral vascular disease, it cannot be used as a long-term alternative to surgical bypass or arterial obstruction removal surgery (82).

Tocopherols, which are commonly known as vitamin E, exist in the forms of α-, β-, γ-, and δ-tocopherol, and can inhibit platelet aggregation, produce nitric oxide in endothelial cells, and produce superoxide in neutrophils and macrophages (83). Since α-tocopherol is a weak antioxidant, it is thought to eliminate the reactive oxygen species involved in the pathogenesis of ORN by inducing cell membrane peroxidation (84). Studies have demonstrated the effectiveness of the combination of pentoxifylline and tocopherol compared with a placebo and drug monotherapy in the treatment of radiation damage in other parts of the body (81, 85, 86).

Clodronate inhibits bone resorption by reducing the number and activity of osteoclasts. Unlike other bisphosphonates, clodronate directly acts on osteoblasts, increasing bone formation and reducing the proliferation of fibroblasts (87, 88). In addition to inhibiting osteoclast activity, clodronate can stimulate osteoblast function and reduce the expression of inflammatory cytokines, which seems to make this drug unique in the bisphosphonate group (87–89). However, the use of the above-mentioned drugs alone is not supported because when used alone, they seem not to affect the condition of ORN (85, 90). Physical therapy, such as extracorporeal shock wave and pulsed electromagnetic field therapy, also plays an important role in restoring movement and improving gait (81, 91).

#### Surgical Management

For patients with advanced ORN or failure of non-surgical treatment, surgical treatment may be considered. For early osteonecrosis, before the articular cartilage is affected and the femoral head is flattened, decompression of the nucleus pulposus of the femoral head is believed to be effective. Although some patients may feel some pain relief after nucleus pulposus decompression, thus far, no well-designed study has found a significant difference in the incidence of femoral head collapse between patients treated with and without nucleus pulposus decompression (92, 93). Indeed, the femoral head collapses rapidly, regardless of whether nucleus pulposus decompression is performed.

Due to bone damage after radiation, THA is usually required in the advanced stages of the disease. However, the loosening rates of cemented and non-cemented standard acetabular cups are very high (44 and 52%, respectively) in patients with ORN (94, 95). Porous tantalum acetabular implants have been found to have potential benefits for acetabular prosthesis fixation. Due to its high coefficient of friction (0.88), high porosity (80%), and high inward growth rate for cancellous bone, the porous tantalum acetabular cup is considered to be capable to undergoing good osseointegration with the surrounding bone (96–98).

However, even with autologous or allogeneic bone transplantation, THA is challenging in patients with ORN (99). Furthermore, for many patients with ORN, initial or multiple total hip replacements are not indicated because of the high postoperative loosening rate, insufficient bone mass, and presence of concomitant diseases (100–102). Girdlestone resection arthroplasty (GRA) is the final choice for end-stage ORN patients who cannot undergo a hip arthroplasty. In the GRA technique, the femoral head is removed, leaving only a rough joint between the femur and the acetabulum. The main goal of GRA is to cure the inflammation and relieve pain (103, 104). GRA is beneficial for patients with severe clinical symptoms, unbearable pain at rest and during work, and severe acetabulum damage. However, limb shortening, hip instability, pseudoarthrosis, the inevitable need for walking aids, and impaired joint function will occur after GRA (105, 106). After undergoing GRA, patients’ ability to perform daily activities is highly restricted, which affects their social life and impacts their mental health. Hence, the choice between GRA and THA combined with an allograft must be carefully discussed with patients. The “salvage” measure of GRA must be used in all cases where there is a local or otherwise unsolvable problem (99).

Compared with ORN of the jaw, there are fewer studies on ORN of the hip. This systematic review comprehensively summarizes the published cases characteristics and related information of ORN of the hip, so as to improve people’s attention and understanding of ORN of the hip. With advancements in modern radiotherapy technology, the radiation dose to normal tissues is being minimized, and important structures and organs are properly protected; these measures are expected to reduce the incidence of radiotherapy-induced vascular disease and minimize the harmful consequences of radiotherapy. But, at present, an evidence-based explanation for the pathogenesis of ORN is lacking, so there is no gold standard for the treatment of hip ORN and no widely accepted guidelines.

The goals of future research on ORN should be to clarify the complex pathogenesis and guide the development of new treatment strategies, to provide safer and more targeted radiation therapy, and to optimize radiation doses so as to kill cancer cells while having a limited impact on normal tissue.

Some limitations of this systematic review should be noted. First, at present, there is no consensus regarding the mechanism of ORN, In the included case studies, most authors diagnose ORN according to the diagnostic prerequisites mentioned in the article, which has a certain subjective intention. Second, the quality assessment tool of case report used in this paper only has the assessment contents, but there is no specific description of the quality score distribution of each item and which parameters are used to divide low, medium and high quality studies. Third, the quality assessment system is easily affected by inter-user variability, especially under the influence of the second limitation. However, two blinded people scored independently, and any disagreements regarding the quality assessment were resolved through discussion and consensus involving a senior reviewer, to maintain the accuracy in present study.

## Conclusion

This systematic review provides a comprehensive summary of the pathogenetic characteristics and knowledge of ORN of the hip. According to our analysis of the characteristics of the included cases, more than half of the included cases were diagnosed with ORN within 4 years after radiotherapy; the primary treatment of hip ORN consists of surgery, as the effects of non-surgical treatments are very limited. Because of the severity of ORN of the hip, and the difficulty in early diagnosis and treatment, clinicians should improve their understanding of this complex disease to prevent it from occurring or progressing to more serious complications.

## Data Availability Statement

The original contributions presented in the study are included in the article/supplementary material, further inquiries can be directed to the corresponding author/s.

## Author Contributions

S-HX and J-LX conceived the article. S-HX wrote the initial draft, reviewed the literature, and edited the manuscript. J-ST and X-YS reviewed and edited the manuscript. Z-XN performed the literature search and provided valuable comments. All authors discussed the results, revised the manuscript, contributed to the article, and approved the final manuscript.

## Conflict of Interest

The authors declare that the research was conducted in the absence of any commercial or financial relationships that could be construed as a potential conflict of interest.

## Publisher’s Note

All claims expressed in this article are solely those of the authors and do not necessarily represent those of their affiliated organizations, or those of the publisher, the editors and the reviewers. Any product that may be evaluated in this article, or claim that may be made by its manufacturer, is not guaranteed or endorsed by the publisher.

## Abbreviations

ORN: Osteoradionecrosis
ESR: Erythrocyte sedimentation rate
CRP: C-reactive protein
WBC: White blood cell
CT: Computed tomography
MRI: Magnetic resonance imaging
T1WI: T1-weighted imaging
T2WI: T2-weighted imaging
THA: Total hip arthroplasty
GRA: Girdlestone resection arthroplasty.
